# Supplementary material for: Advanced Glycation End Products and Their Effect on Vascular Complications in Type 2 Diabetes Mellitus
Source: Nutrients. 2022 Jul 27;14(15):3086. doi: 10.3390/nu14153086 (PMC9370094; doi:10.3390/nu14153086)
Supplement: Supplementary file 1 [file nutrients-14-03086-s001.zip › nutrients-1821531-supplementary.pdf]

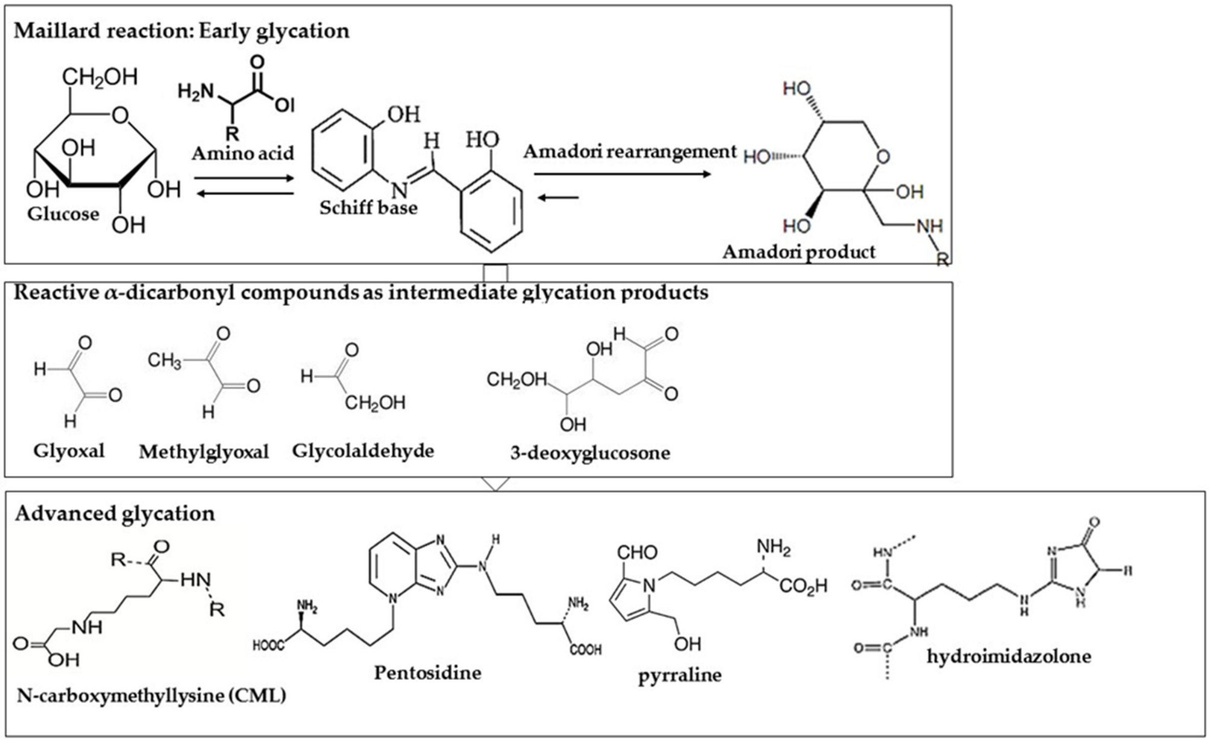

Figure S1. Maillard reaction and advanced glycation end products (AGEs) formation: The Maillard reaction is initiated with the formation of a Schiff base and an Amadori product. After classic rearrangement, the oxidative pathway, or the non-oxidative pathway, AGEs are generated.
